# First person – James D. Hurcomb and Amrita Mukherjee

**DOI:** 10.1242/dmm.052340

**Published:** 2025-03-24

## Abstract

First Person is a series of interviews with the first authors of a selection of papers published in Disease Models & Mechanisms, helping researchers promote themselves alongside their papers. James D. Hurcomb and Amrita Mukherjee are co-first authors on ‘ Oral administration of aripiprazole to *Drosophila* causes intestinal toxicity’, published in DMM. James is a PhD student in the lab of L. Miguel Martins at ​the MRC Toxicology Unit, University of Cambridge, Cambridge, UK, investigating inter-organ communication and whole-body metabolic response in response to mitochondrial toxicity. Amrita is a research fellow in the same lab and is interested in mitochondrial toxicity and the effect it has in different tissues and on cellular metabolism.


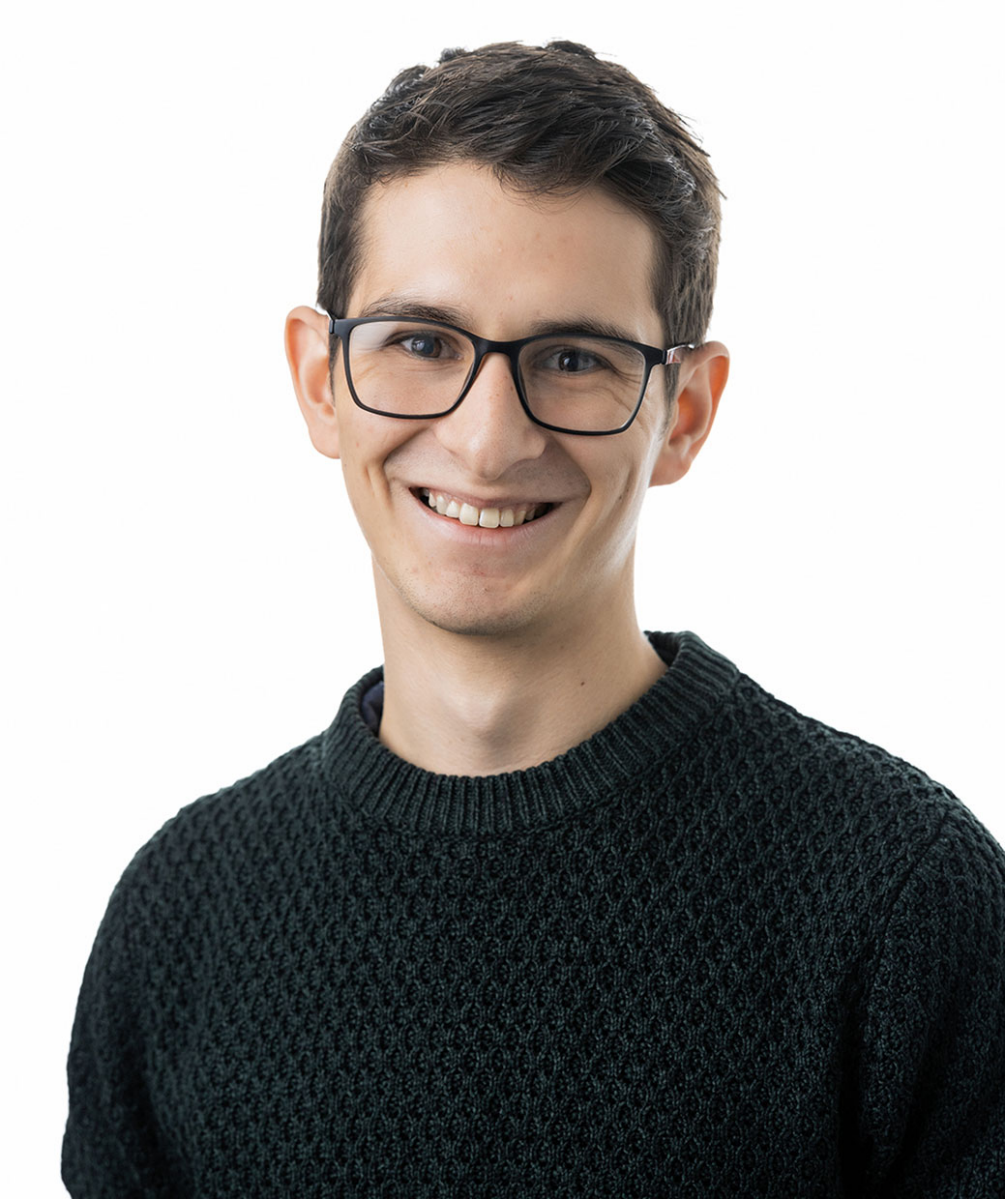


**James D. Hurcomb**

**Who or what inspired you to become a scientist?**

**J.D.H.:** I was always interested in science and nature from a young age and was fortunate to have some great biology and chemistry teachers throughout my education. However, despite studying a biomedical science degree, I wasn't set on becoming a scientist until I took part in two research projects as an undergraduate working on fruit flies. The fruit flies (or, more so, the professors and scientists working on them) inspired me to become a scientist!

**A.M.:** I think it was a combination of a number of factors that led me on this path. My earliest memory of wanting to become a scientist was when I received a book on astronomy, and I was absolutely fascinated by the universe. Although, later I got interested in genetics and cell biology and took these up as my topic of studies. Working as a scientist is like solving a mystery or a puzzle. It often starts as a curiosity and one cannot really stop until it is figured out.

Drugs taken in orally can have an off-target impact on gut epithelial cells, and, as a result, patients often show gastrointestinal side effects.

**What is the main question or challenge in disease biology you are addressing in this paper? How did you go about investigating your question or challenge?**

**J.D.H.:** All medicines have side effects, and one of the most common places for side effects to happen for medicines that are taken by mouth is in the intestine, even if the drug isn't meant to treat an intestinal disease. Relatively little research has been conducted on what causes these effects. We investigated how these side effects are caused by aripiprazole using a fruit fly model system. Fruit flies are surprisingly similar to humans in some ways – sharing many genes and even a similar gut structure – making them an ideal, low-cost, simple model to answer this question.

**A.M.:** Mitochondria are fascinating organelles and sit at the centre of cellular biochemistry. Many drugs tend to have a detrimental off-target effect on mitochondria inside cells. Drugs taken in orally can have an off-target impact on gut epithelial cells, and, as a result, patients often show gastrointestinal side effects. Understanding the mechanism of toxicity of such orally administered drugs (in this case, aripiprazole, a commonly prescribed antipsychotic medication) is crucial to find ways to mitigate some of these undesired side effects. We used a fruit fly model to investigate the mechanism of the off-target effect of orally administered aripiprazole on gut epithelial cells.

**How would you explain the main findings of your paper to non-scientific family and friends?**

**J.D.H.:** We investigated how the common antipsychotic drug aripiprazole impacted the intestine by feeding the drug to fruit flies. We found that, in flies, as in humans, aripiprazole causes gastrointestinal side effects. Aripiprazole can inhibit the ability of cells to generate energy using their mitochondria. This inhibition causes production of reactive chemicals called reactive oxygen species, which damage the intestine, causing side effects and ultimately leading to the death of cells in the gut, triggered by a pathway that is also present in humans. We found that feeding the flies with an antioxidant (to mop up the toxic reactive oxygen species) alongside aripiprazole significantly reduced the damage to the intestine.

**A.M.:** Every cell needs to produce energy, and this function is carried out by a special tubular network of subcellular structures called mitochondria. We found that a commonly prescribed antipsychotic medication, aripiprazole, damages the mitochondria of gut epithelial cells, resulting in their death, in a fruit fly model. This damage was mitigated by providing antioxidant supplements along with the medication.

**What are the potential implications of these results for disease biology and the possible impact on patients?**

**J.D.H.:** We found that, in our model system, feeding flies antioxidants was enough to significantly reduce the severity of gastrointestinal side effects of the drug. It is possible that, in the future, antioxidants could be prescribed alongside drugs with known off-target impacts on mitochondria to reduce side effects. However, much more research is needed before this can reach patients!

**A.M.:** Our findings showed that the off-target mitochondrial damage caused by the antipsychotic medication results in gastrointestinal dysfunction. This can be reduced significantly by supplementing the fly diet with antioxidants. Although this requires further investigation, our study implies that there is a non-invasive possibility of mitigating the gastrointestinal side effects of orally administered antipsychotic medication for patients.


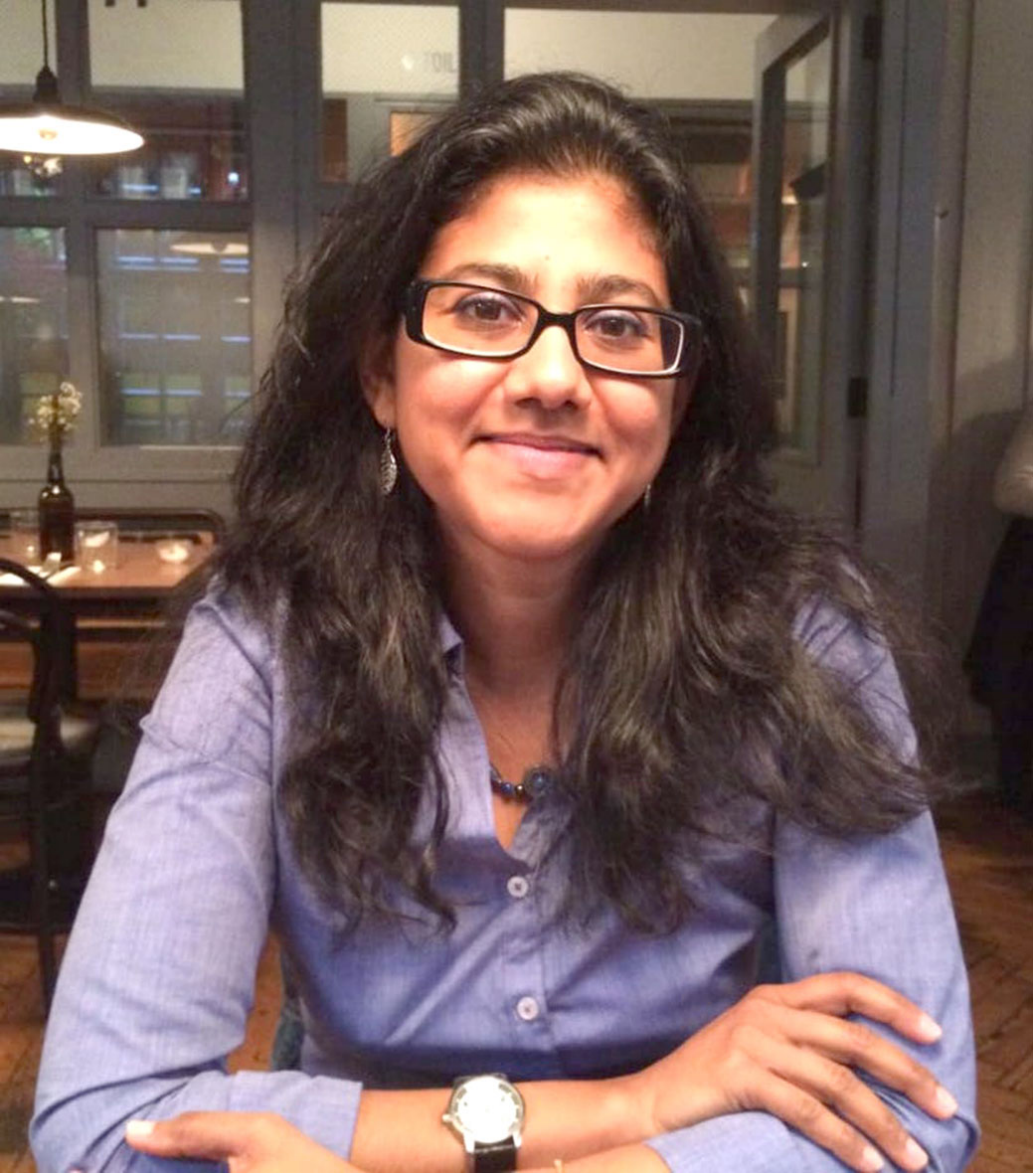


**Amrita Mukherjee**

**Figure:**
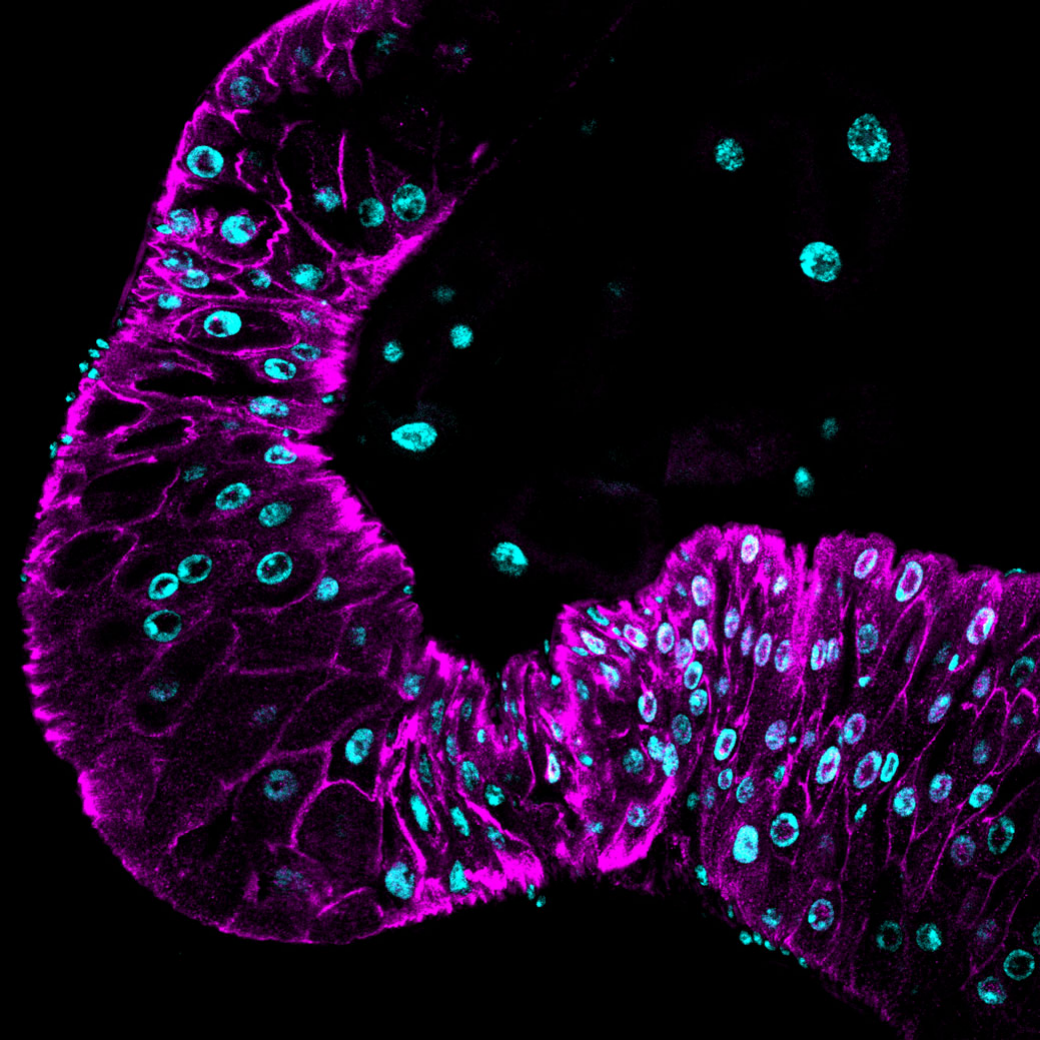
Midgut of *Drosophila melanogaster* supplemented with aripiprazole, showing the cell death marker Dcp-1 (magenta) and nuclei (cyan).

**Why did you choose DMM for your paper?**

**J.D.H.:** We chose DMM for our paper as it's a well-respected journal that publishes interesting science about new models for investigating diseases. Also, we love that they plant a tree for every paper they publish!

**A.M.:** Our study focuses on understanding the mechanism of the off-target effect of a commonly prescribed antipsychotic medication. DMM, an Open Access journal aiming to advance novel insights into the mechanism and diagnosis of human diseases, was most suitable for our findings.

**Given your current role, what challenges do you face and what changes could improve the professional lives of other scientists in this role?**

**J.D.H.:** The professional life of a PhD student is always challenging, with pressures coming from many directions, but I've been lucky to work in a supportive environment during my PhD. A particular concern I have for other scientists at a similar career stage in the UK is the recent change to the funding of MRC Research Units. The decision to eliminate Units for shorter-term investments means that future generations of scientists won't be able to benefit from the stable high-quality training that I received. For early-career scientists, stability and high quality of training is essential.

**A.M.:** As a postdoctoral researcher/research fellow, I believe that the biggest challenges are short-term contracts of these positions. The uncertainty of future contracts does not only have a negative impact on the mental health and personal lives of the researchers, but it also prevents early-career scientists from investing in risky and innovative projects. Short-term contracts generally breed incremental science, which, in the long run, is a loss for the general scientific community and, in some sense, also a waste of public funding.

**What's next for you?**

**J.D.H.:** I'm in the final year of my PhD, so I am working on tying up some other projects and writing my thesis. After that, I hope to take what I've learnt over the last few years and apply it to interesting new problems.

**A.M.:** My current research position is as a research fellow. I am looking to obtain independent funding to venture into my own research directions.

**Tell us something interesting about yourself that wouldn't be on your CV**

**J.D.H.:** Outside of work, I spend a lot of time reading, running and swimming.

**A.M.:** I am a science fiction aficionado. In my spare time, I also enjoy doing ink and watercolour paintings and learning to play the violin.
